# Homeostatic regulation in a single neuron model from the Pre-Bötzinger Complex

**DOI:** 10.1186/1471-2202-13-S1-P158

**Published:** 2012-07-16

**Authors:** Max F Oginsky, Gennady S Cymbalyuk

**Affiliations:** 1 Department of Biology, Georgia State University, Atlanta, GA 30303, USA; 2 Neuroscience Institute, Georgia State University, Atlanta, GA 30303, USA

## 

Central Pattern Generators (CPGs) control rhythmic movements in diverse species ranging from decapods to mammals. For the same CPG, from preparation to preparation, the intrinsic membrane properties of CPG neurons are highly disparate and modulated, however the network output remains constant*.* This leads to questions regarding the coregulation of currents preserving essential dynamics of a neuron. When the fast-transient potassium current, I_A_, is increased in the pyloric dilator neuron of the stomatogastric ganglion, there is a compensatory increase in the hyperpolarization-activated current, I_H_[1]. This prevents changes in the output of this well-studied CPG. Here, we test whether this coregulation would be effective if applied to the dynamics of another well-studied CPG found in mammals that controls breathing. We hypothesized that when the maximal conductance, *ḡ_A_*, of I_A_ was increased there would be an increase in the period and interburst interval (IBI) and subsequently increasing the maximal conductance, *ḡ_H_*, of I_H_ would provide a matching compensatory decrease. To investigate this, we modified a single neuron model of the pre-Bötzinger complex [2] by adding I_A _[3] and I_H_[4]. We investigated this model by systematically exploring properties of the ionic currents.

**Figure 1:**
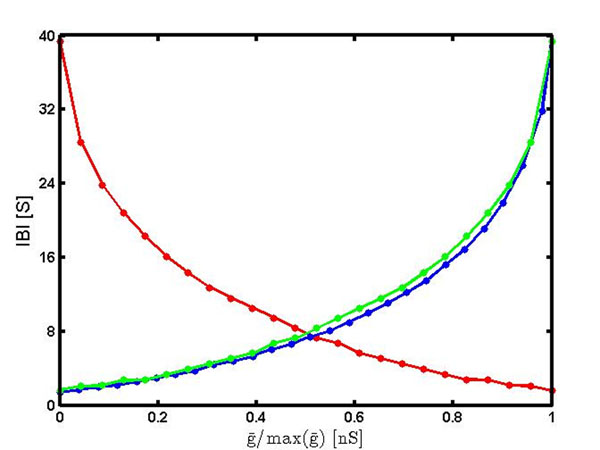
Dependence of interburst interval on *ḡ_A_* (blue) and *ḡ_A_* (red). Conductances were normalized to their maxima, max(*ḡ_A_*) and max (*ḡ_H_*). Green curve represents the IBI dependence on .

First, we increased *ḡ_A_* from 0 to 51nS. This increased the period and IBI from 1.78s to 40.12s and 1.37s to 39.35s, respectively (Fig. 1, blue curve). At the same time, the spike frequency and spike number increased from 10.29 to 60.72Hz and 5.00 to 38.35 spikes, respectively. Then we subsequently increased *ḡ_H_* from 0nS to 69nS. The period and IBI was decreased from 40.12s to 2.02s and 39.35 to 1.64s, respectively (Fig. 1, red curve). The spike frequency and spike number decreased from 60.72 to 13.93Hz and 36.50 to 2.74 spikes, respectively. In Figure 1 we presented the data for the IBIs. To place the graphs on the same scale we normalized the parameter values by the maximum values used in the study. The blue and red curves look similar, except *ḡ_A_* graph grows and *ḡ_H_* graph decays. To further evaluate how well the two dependences are matched, we plotted the IBI dependence on  as the green curve. One could see that the green and blue curves are well matched.

This model shows that increasing I_H_ opposes the effects of increasing I_A_ in period, IBI, frequency and spike number. This may be a common mechanism for regulating rhythmic patterns in CPGs.
